# Supplementary material for: Protein Biocargo and Anti-Inflammatory Effect of Tomato Fruit-Derived Nanovesicles Separated by Density Gradient Ultracentrifugation and Loaded with Curcumin
Source: Pharmaceutics. 2023 Jan 19;15(2):333. doi: 10.3390/pharmaceutics15020333 (PMC9961453; doi:10.3390/pharmaceutics15020333)
Supplement: Supplementary file 1 [file pharmaceutics-15-00333-s001.zip › Supplementary Figure S1.pdf]

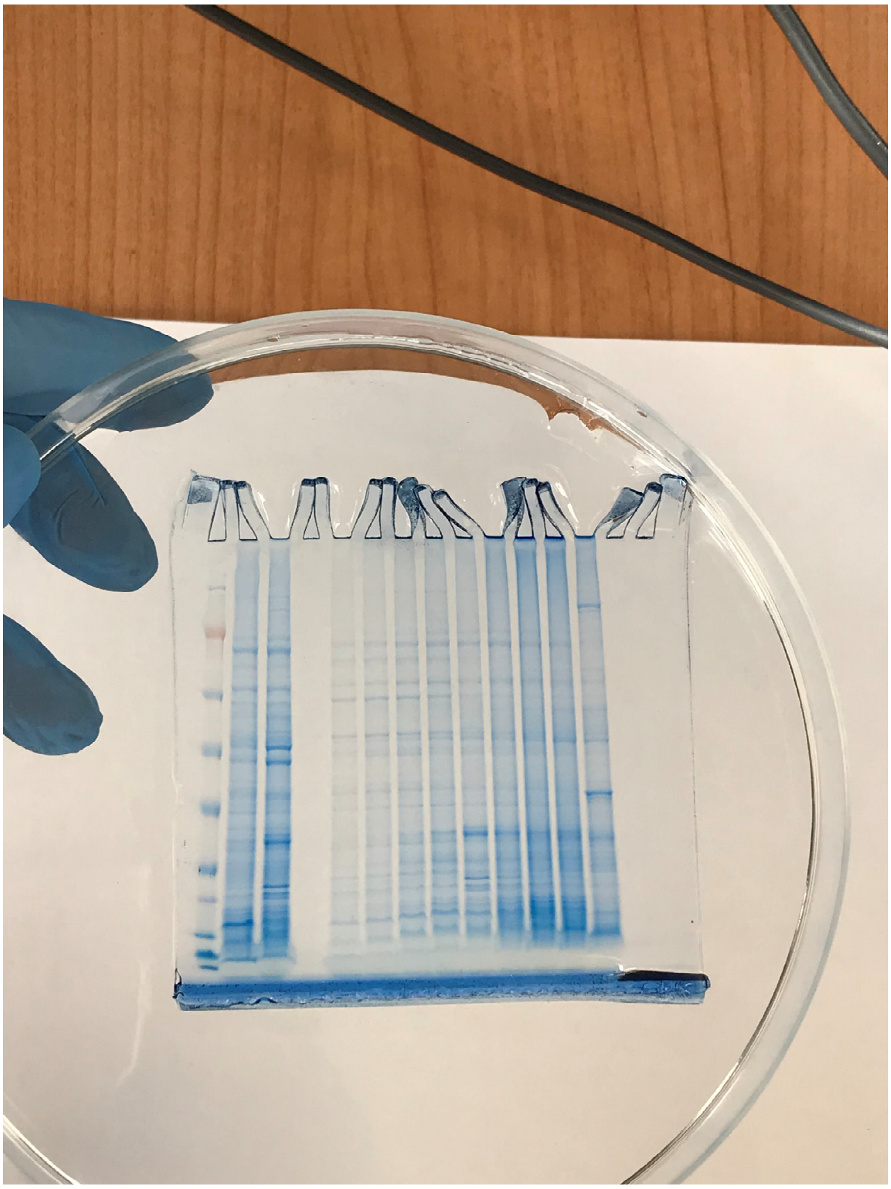

Figure S1. Uncropped sodium dodecyl sulfate–polyacrylamide gel electrophoresis (SDS-PAGE) image of Figure 2c. Protein profiles of tomato NVs and fractions separated by iodixanol DGUC (the loaded protein quantity for each fraction was 10 µg, except Fraction 1, 11 and 12, for these fractions the protein amount was around 1 µg).
